# Trauma and PTSD: setting the research agenda

**DOI:** 10.3402/ejpt.v6.28092

**Published:** 2015-05-19

**Authors:** Miranda Olff

Up to 80% of people encounter severe adverse events in their lives (De Vries & Olff, 2009). Most people will be resilient or quickly recover from negative symptoms, but a significant proportion will develop posttraumatic stress disorder (PTSD) leading to a lifetime prevalence of PTSD in about 7% (De Vries & Olff, 2009; Kessler et al., 2005). These events precipitate not only PTSD but also major depression, anxiety disorders, addiction, physical health problems, and other trauma-related disorders. Early and repeated trauma, such as child abuse or interpersonal violence, has been associated with long-term negative outcomes (Olff & Wall, 2014). Complex PTSD (CPTSD), which is a separate diagnosis for International Classification of Diseases (ICD-11) and has many of its symptoms now incorporated in the Diagnostic and Statistical Manual (DSM-5) diagnosis of PTSD, is highly prevalent in clinical practice. Lack of recognition of mental health conequences of trauma or under-treatment is not only likely to result in chronic disorders but also in significant costs to society (“PTSD care, out of service”, 2014).

In short, PTSD and other trauma-related negative outcomes are highly prevalent and debilitating consequences of trauma. Yet, we still know little about their hallmark symptoms and symptom structure, their underlying neurobiology, their (subclinical) expression, or about biases in reporting. There is thus a very strong rationale to study and understand the symptoms, profiles, and mechanism of traumatic stress responses, and to optimize prevention and treatment of trauma-related disorders. This special issue will address the gaps in our knowledge and point to novel research directions.

With this special issue, we celebrate the 5th year of *European Journal of Psychotraumatology*. The journal was launched in December 2010 (Olff, 2010) and developed into a respected journal indexed in major databases over the past years (Olff, 2012, 2013, 2014; Olff & Bindslev, 2011), including the Social Science Citation Index and Scopus. The journal is open to new developments and invites contributions from the different “schools” for scientific debate. With this issue, we aim to stimulate research and to move ahead in the field, hopefully leading to much needed improvements in science and practice with regard to those suffering from the consequences of severe trauma.

The editors of the journal have joined forces to summarize the state of the art in their specific areas of expertise, identify the knowledge gaps, and point out research directions. The authors address basic features of traumatic stress responses such as flashbacks (Brewin, 2015) and the structure of PTSD (Armour, 2015), subthreshold PTSD (Schmidt, 2015), the “perception” of disorder in the legal worlds (Herlihy & Turner, 2015), trauma-related dissociation, altered states of consciousness and their neurobiological substrate (Lanius, 2015), and how early and repeated life adversities may lead to CPTSD, both in youngsters (Ford, 2015) and adults (Cloitre, 2015). Important research suggestions are made with regard to treatment of complex forms of PTSD for children and adolescents (Ford, 2015) and adults (Cloitre, 2015), treatment of prolonged or complicated grief (Rosner, 2015), and, finally, with regard to new forms of interventions with mobile apps (Olff, 2015).

In his contribution, Brewin (2015) focuses on posttraumatic flashbacks. It is a hallmark symptom of PTSD both in the DSM-5 and in the proposed ICD-11, yet little research into flashbacks has been conducted. Brewin is showing us what type of research efforts are required to understand the cognitive and biological basis of this important symptom and discusses assessment of flashbacks. Research in other contexts, such as psychosis or intensive care, is another area of interest.

Which latent model of PTSD best represents PTSD's underlying dimensionality, is the question Armour (2015) addresses. She provides a clear rationale as to why research into the structure of PTSD is a pertinent research area, reviews the literature pertaining to the DSM-IV and DSM-5, and provides recommendations for future research directions.

Schmidt (2015) addresses the significant proportion of patients suffering from subthreshold diagnoses, such as partial PTSD, who present in clinical practice and how use of today's traditional diagnostic system in psychiatric research does not sufficiently promote an integrative understanding of mental disorders across multiple units of analysis from behavior to neurobiology. She embraces the RDoC methodology and suggests enriching it with symptom-oriented research.

Lanius (2015) describes trauma-related dissociation and altered states of consciousness in the context of a 4-dimensional model. This fascinating conceptualization across the dimensions of time, thought, body, and emotion has transdiagnostic implications for trauma-related disorders described in both the DSM and ICD. The 4-dimensional model provides a framework, guided by existing models of dissociation, for future research examining the phenomenological, neurobiological, and physiological underpinnings of trauma-related dissociation.

With the aim of limiting the inappropriate reliance on assumptions and stereotypes, and advocating for the greater use of psychological science, in legal decision-making, Herlihy and Turner (2015) outline and discuss the contribution that research on trauma and related psychological processes can make to two particular areas of law where difficult legal decisions must be made: in claims for refugee and humanitarian protection, and in reporting and prosecuting sexual assault in the criminal justice system.

Ford (2015) discusses research directions in CPTSD and developmental trauma disorder (DTD) in children and adolescents addressing diagnostic classification and psychometric assessment, interventions, and the epidemiology as well as their public health and safety impact across the lifespan.

In her article, Cloitre (2015) provides arguments for the recognition of the heterogeneity of symptoms in trauma populations (such as PTSD vs. CPTSD) and the development of treatments that promote the tailoring of interventions according to patient needs. New research methodologies are proposed that can incorporate important variables such as patient preferences and symptom heterogeneity without necessarily extending already lengthy study times or further complicating study designs.

Rosner (2015) outlines findings and defines important areas for future research with regard to prolonged grief disorder, as proposed by ICD-11, viewed from a lifespan perspective. Rosner pleads for valid psychometric measures for the new diagnosis and specifically so for children and adolescents, and how treatments need to be adapted for specific subgroups. She also points out the need to disseminate the available knowledge about prolonged grief into various clinical and non-clinical settings.

Olff (2015) describes the exciting developments in the field of mobile health (“m-Health”) applications (apps) targeting screening, assessment, prevention, and treatment. While there is an explosive growth of tools on the market, few of the mobile apps have been rigorously evaluated. But how feasible is rigorous scientific evaluation with the rising demands—from policy makers, business partners, and users—for their quick release? Evidence-based guidance is suggested for appropriate research designs, which may overcome some of the public and ethical challenges (e.g., equity, availability) and the market-driven wish to have mobile apps in the “App Store” yesterday rather than tomorrow.

As mentioned above, this cluster of articles is based on contributions from the *EJPT* editors, but there are of course many other experts in the field who may have valuable ideas about the research agenda for the study of trauma and its consequences. You are more than welcome to submit a paper to this cluster. Many of us have ideas for future studies and numerous grant applications are being written every year; the more focus *we* have on the research agenda and the better we can present it, the more it will hopefully shape *funding bodies’* thoughts about the focus of future research.

*Miranda Olff* Editor-in-ChiefAssociate Editors *Cherie Armour* *Chris Brewin* *Marylene Cloitre* *Julian D. Ford* *Jane Herlihy* *Ruth Lanius* *Rita Rosner* *Ulrike Schmidt* *Stuart Turner*
